# Incidence and Impact of Patient-Prosthesis Mismatch in Isolated Aortic Valve Surgery

**DOI:** 10.3889/oamjms.2015.108

**Published:** 2015-10-15

**Authors:** Selman Dumani, Ermal Likaj, Andi Kacani, Laureta Dibra, Elizana Petrela, Vera Beca, Ali Refatllari

**Affiliations:** 1 *Service of Cardiac Surgery, University Hospital Center “Mother Theresa”, Tirana, Albania*; 2 *Service of Statistics, University Hospital Center “Mother Theresa”, Tirana, Albania*; 3 *Obstetrics and Gynecology Hospital “Queen Geraldine”, Tirana, Albania*

**Keywords:** aortic valve, patient-prosthesis mismatch, valve prosthesis

## Abstract

**AIM:**

The mains topics of this work are the incidence of patient-prosthesis mismatch and the influence in the early results of isolated aortic valve surgery.

**METHODS:**

In 193 patients isolated aortic valve surgery was performed. The study population was divided in three subgroups: 20 patients with severe, 131 patients with moderate and 42 patients without patient-prosthesis mismatch. The indexed effective orifice area was used to define the subgroups. Operative mortality and perioperative complications were considered the indicators of the early results of aortic valve surgery.

**RESULTS:**

The incidence of severe and moderate patient-prosthesis mismatch was respectively 10.3% and 67.8%. Hospital mortality and perioperative complications were: mortality 5% vs. 3.1% vs. 2.4% (p = 0.855), low cardiac output 5% vs. 6.9% vs. 4.8% (p = 0.861); pulmonary complications 5% vs. 3.1 vs. 0.0% (p = 0.430); exploration for bleeding 5% vs. 0.8% vs. 2.4% (p = 0.319); atrial fibrillation 30% vs. 19.8% vs. 11.9% (p = 0.225); wound infection 5% vs. 0.8% vs. 0.00% (p = 0.165), respectively for the group with severe, moderate and without patient-prosthesis mismatch.

**CONCLUSIONS:**

Patient-prosthesis mismatch is a common occurrence in aortic valve surgery. This phenomenon does not affect the early results of aortic valve surgery.

## Introduction

Patient prosthesis mismatch (PPM) is one of the most controversial issues related to the aortic valve replacement. It is introduced in 1978 by Rahimtoola as a condition in which the effective prosthetic valve area, after insertion into the patient, is less than that of a normal human valve [1]. There are two main occurrence factors of PPM:

a) Effective orifice area “in vivo” and “in vitro” of the prosthesis; and
b) The patient’s small native valve annulus.

In practical terms, to define PPM currently, the most used parameter is prosthetic effective orifice surface area indexed with the patient’s body surface (EOAi). Based on this index, threshold for the appearance of PPM is 0.85 cm^2^/m^2^. Patient prosthesis mismatch has been classified as moderate when 0.65 cm^2^/m^2^ ≤ EOAi ≤ 0.85cm^2^/m^2^ and severe when EOAi < 0.65 cm^2^/m^2^.

Patient-prosthesis mismatch is a common occurrence after aortic valve replacement. The reported prevalence of moderate PPM varies between 20–70%, whereas that of severe PPM is between 2% and 11% [2]. Nowadays, the discussion is still open about the impact of this phenomenon in the results of this kind of surgery.

The mains topics of this work are the incidence of patient-prosthesis mismatch in aortic valve surgery and the influence of these findings in the early results.

## Materials and Methods

Our study was a prospective and retrospective study. All patients underwent isolated aortic valve surgery from January 2010 to January 2015 in two cardiac surgery centres, public and non-public, in Tirana (Service of Cardiac Surgery, UHC “Mother Theresa” and German Hospital). Patients who had additional surgical procedures were excluded from the study. Demographic data, intervention and postoperative data were collected from hospital records in the statistics office. Preoperative evaluation of risk of intervention was made according to EUROSCORE [5]. Surgical indication for aortic valve surgery was decided according to ESC and AHA/ACC guidelines [6, 7]. Indexed effective orifice area was used to define the degree of PPM. The effective orifice area of the prosthesis was taken from the reference prosthesis tables [2-4] while BSA was calculated according Du Bois and Du Bois formula. The body mass index was also recorded, which was calculated according to the metric formula (kg/m^2^). Hospital mortality and major complications such as low cardiac output, cerebral accidents, pulmonary complications, renal disorders, wound infection etc. were considered end-points for the evaluation of early results of aortic valve surgery.

### Surgical Procedure

A full midline sternotomy was performed. After establishing cardiopulmonary bypass and clamping the aorta, myocardial protection was provided by intermittent antegrade cardioplegia. We used to do the first dose crystalloid and after warm blood cardioplegia; transverse aortotomy; resection of the native aortic valve cusps and meticulous decalcification of the aortic annulus. All prosthesis were implanted with mattress technique, using double-armed, pleged-suported TiCron 2/0 sutures

The heart was de-aired and the aortic clamp removed after the closure of the aorta. At the end of the operation the cannulas was removed and protamine was given. Temporary pacemaker (PM) wires, mediastinal, and pleural tubes were placed before chest closure.

### Statistical analysis

Continuous data were presented at the mean value and standard deviation. Discrete data were presented in absolute value and percentage. Differences between the two groups for continuous quantitative variables were performed by ANOVA test one way, the Bonferoni procedure. Differences between groups for discrete variables were performed by Chi-Square test. Relations between variables were analyzed through Pearson correlation coefficient (for quantitative variables) and Kendall’s Tau (for discrete variables). Presentation of the data was performed through simple and composed tables, as well as graphics like scatter diagram, bar-diagram and Box-plott. Data analysis was performed with SPSS statistical package, version 20, (Statistical Package for Social Sciences). It was considered significant values of p ≤ 0.05.

## Results

### Patients

In the study were included 193 patients that performed isolated aortic valve surgery. Patients were divided into three groups according to the degree of PPM: 20 patients with severe PPM; 131 patients with moderate PPM; and 42 patients without PPM.

General demographic and clinical data are presented in Table 1.

**Table 1:** General demographic and clinical data

General Data		Groups PPM		
		
		MPP severe n=20	MPP moderate n=131	No MPP n=42	Total 193	Value p
Age		63.55**±**11.97	60.47±12.47	54.69±12.83	59.53±12.73	0.012

BMI		27.26±3.75	25.01±3.27	23.84±3.47	24.99±3.47	0.01

BSA		1.85±0.13	1.79±0.16	1.75±0.20	1.79±0.17	0.10

Gender	M	9	82	33	124

		45.00%	63.10%	78.60%	64.60%

	F	11	48	9	69	

		55.00%	36.90%	21.40%	35.40%	0.029

Admission	urgent	0	7	4	11	

		0.00%	5.30%	9.50%	5.70%	

	planed	20	123	38	181	

		100.00%	93.90%	90.50%	93.80%	0.583

NYHA	II	6	32	6	45	

		30.00%	24.60%	14.60%	23.00%	

	III	12	90	34	137	

		60.00%	69.20%	82.90%	71.20%	

	IV	2	8	1	11	

		10.00%	6.20%	2.40%	5.80%	0.345

HT		16	85	20	121	

		80.00%	64.90%	47.60%	62.70%	0.032

DM		6	14	1	21	

		30.00%	10.70%	2.40%	10.90%	0.005

Ap		1	1	0	2	

		5.00%	0.80%	0.00%	1.00%	0.165

Smoking		4	15	10	29	

		20.00%	11.50%	23.80%	15.00%	0.12

COPD		1	5	0	6	

		5.00%	3.80%	0.00%	3.10%	0.406

Esc Stand		4.90±1.66	4.23±2.19	4.03±2.33	4.23±2.18	0.554

Esc Log		4.37±2.90	4.14±4.23	4.10±4.33	4.15±4.13	0.984

BMI-body mass index; BSA-body surface area; HT-hypertension; DM-diabetes mellitus; AP-peripheral artheriopathi; COPD-chronic obstructive pulmonary diseases; Esc- Stand/Log-euro score standard/logistic.

The mean population’s age was 59.53 ± 12.73. Groups with PPM had significant higher mean age comparing to other groups; 124 patients (64.6%) were males. The incidence of co morbidities was without significant statistical differences among groups except diabetes mellitus and arterial hypertension that predominate in the group with the moderate PPM. BMI was importantly greater in groups with PPM. There were no significant differences in the preliminary calculation of intervention risk.

Transthoracic echocardiography and transesophagical echocardiography were the main diagnostic tools. There were 121 patients (62.7%) with pure aortic stenosis; 44 patients (22.8%) with aortic insufficiency and the rest of the patients (had mixed pathology of aortic valve (28 pt. 14.5%)). Echocardiography data were presented in the Table 2.

**Table 2:** Echocardiography data

Data		PPM severe	MPP moderate	No MPP	Total	Value p
EF		62.41±8.12	59.86±10.37	59.98±10.26	60.14±10.12	0.623

Anatomy	Normal	18 (90%)	100 82.6%)	32 80%	156 82.9%	

	Bicuspid	2	21	8	37	

		10.00%	17.40%	20.00%	17.10%	0.621

Ao Val	Stenotic	18	83	20	121	

		90.00 %	63.36 %	47.62 %	62.69 %	

	Regurgit	1	28	15	44	

		5.00%	21.37%	35.71%	22.80%	

	Mixed	1	20	7	28	

		5.00%	15.27%	16.67%	14.51%	0.023

Calcified Annulus		17 85.00%	84 66.7%	20 51.3%	121 65.4%	0.031

ThPW		12.82±0.99	13.21±2.02	12.53±1.98	13.01±1.94	0.222

ThS		14.00±1.11	14.39±2.15	13.53±2.18	14.15±2.10	0.129

LVTDD		53.75±6.77	57.59±8.20	59.70±8.76	57.74±8.31	0.107

LVTSD		34.08±7.14	39.96±9.65	39.34±8.40	39.15±9.19	0.123

Max Val grad		87.67±16.78	88.78±20.20	88.41±18.58	88.57±19.31	0.983

Mean Val grad		48.31±8.84	52.45±13.77	55.65±7.41	52.43±12.55	0.220

EF-ejection fraction; Ao Val-aortic valve; ThPW-thikness of posterior wall; ThS-thikness of septum LVTDD-left ventricle telediastolic diameter; LVTSD-left ventricle telesistolic diameter; Max Val grad-maximal aortic valve gradient; Mean Val grad-mean aortic valve gradient

The echocardiography data showed that aortic stenosis was the dominant pathology (121/193 pt, 62.7 %) in our population and annular calcification is encountered much more often in the PPM groups.

Incidence of patient-prosthesis mismatch: incidence of severe PPM was 10.3% (20/193); and incidence of moderate PPM was 67.8% (131/193).

Early mortality and postoperative complications: overall mortality was 3.1% (6/193); mortality of the group with severe PPM was 5% (1/20 pt); mortality of the group with moderate PPM was 3.1% (4/131 pt); and mortality of the group without PPM was 2.4 % (1/42 pt).

We can see that in absolute value, mortality in the groups with PPM is higher but this difference is not statistically significant (p = 0.855). The incidence of postoperative complications respectively for the group with severe PPM, moderate and without PPM was: low cardiac output 5% vs. 6.9% vs. 4.8%; pulmonary complications 5% vs. 3.1 vs. 0.0%; bleeding 5% vs. 0.8% vs. 2.4%; atrial fibrillation 30% vs. 19.8% vs. 11.9%; wound infection 5% vs. 0.8% vs. 0.00%; ventricular arrhythmias 5 % vs. 4.6 % vs. 9.5 %; conduction disturbances 10% vs. 6.9 % vs. 11.9 %

There is a trend towards higher frequency of complications in the groups with PPM but the differences did not reach statistical significances (Table 3).

**Table 3:** Mortality and postoperative complications

Morality and complications	Groups PPM				Value p
	Total n=193	PPM severe n=20	PPM moderate n=131	No PPM n=42	
Mortality	1	4	1	6	0.855
	5.00%	3.10%	2.40%	3.10%
LCO	1	9	2	12
	5.00%	6.90%	4.80%	6.20%	0.861
Stroke	0	1	0	1
	0.00%	0.80%	0.00%	0.50%	0.788
Pulmonary	1	4	0	5
	5.00%	3.10%	0.00%	2.60%	0.430
RI	0	1	1	2
	0.00%	0.80%	2.40%	1.00%	0.593
Bleeding	1	1	1	3
	5.00%	0.80%	2.40%	1.60%	0.319
VA	1	6	4	11
	5.00%	4.60%	9.50%	5.70%	0.480
AF	6	26	5	37
	30.00%	19.80%	11.90%	19.20%	0.225
Wound inf	1	1	0	2
	5.00%	0.80%	0.00%	1.00%	0.165
Cond Dist	2	9	5	16
	10.00%	6.90%	11.90%	8.30%	0.564

LCO-low cardiac output; RI-renal insuficiency;VA-ventricular arrhythmias; AF-atrial fibrillation inf-infection;Cond Dist-conduction disturbances.

Postoperative times in intensive care unit and hospital stay are longer in the group with PPM but the difference is not important. The mean prosthesis number used is 21.59 ± 1.88. It is clear the significant difference of mean prosthesis number between groups (p < 0.001). The maximal prosthesis gradient of the PPM groups are much higher in comparison with the group without PPM (p = 0.022) (Table 4).

**Table 4:** Intervention and postoperative data

Intervention and postoperative data		PPM severe n=20	PPM moderate n=131	No PPM n=42	Total	Value p
CPB		92.21±19.42	86.54±24.67	92.03±23.58	88.2930±23.93	0.379
IT		78.67±23.57	68.93±22.42	66.77±21.84	9.43±22.51	0.163
Resp Ass		42.60±110.74	19.89±57.37	11.26±7.98	20.41±59.53	
ICU Stay	(h)	97.9±13.9	65.83±10.1	51.14±26.24	65.96+9.6	0.202
Hos Day	(d)	11.65±7.25	9.82±5.66	10.54±7.73	10.16+6.32	
Prot Nr		19.20±0.62	21.40±1.46	23.33±1.92	21.59±1.88	<0.001
Max Prot grad		33.50±11.8	27.91±9.89	23.69±8.2	27.34±9.97	0.022

CPB-cardio pulmonary bypass; IT-ischemic time; Resp Ass-respiratory assistance; ICU –intensive care unit; Hos –hospital; Prot Nr-prosthesis number; Max Prot grad-maximal transprosthetic gradient

The following diagrams show correlation of PPM with the patient’s age, BMI, BSA, the number of prosthesis, gender.

**Figure 1:**
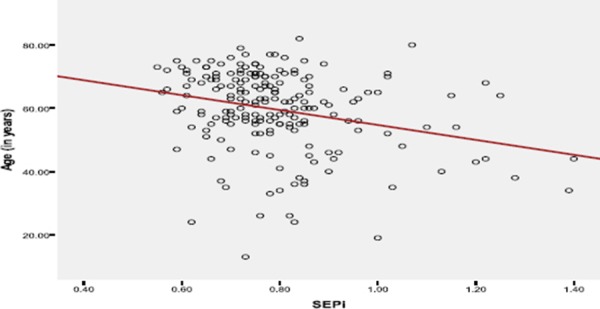
*Correlation between age and the possibility of PPM occurrence. We have an inverse relation between age and EOAi (EOAi=SEPi)*.

## Discussion

This is the first time that the incidence of PPM in aortic valve surgery has been reported in Albania. It is 10.3% for severe PPM and 67.8% for moderate PPM. Pibarot and Dumesnil refers that the incidence of severe PPM varies 2-11 % whereas that of moderate PPM 20-70% [2]. In separate studies, the incidence of severe and moderate PPM is referred respectively 2.3% -6.8% and 8.5% -58.7% [8-14].

**Figure 2:**
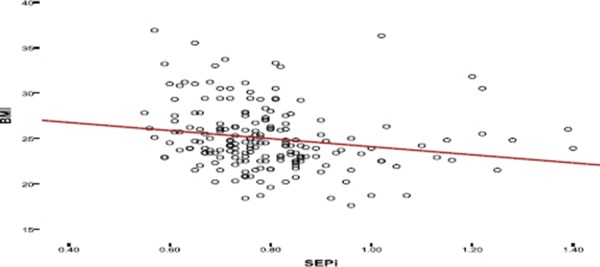
*Correlation between PPM and BMI. Greater BMI - higher the possibility of PPM occurrence*.

We note that EOAi values that are taken as point references for the presence or not of PPM were different in various studies and they range from 0.7-0, 85cm^2^ / m^2^. In our study we used traditional point of references: 0.65cm^2^ / m^2^ ≤EOAi≤0.85cm^2^ / m^2^ for moderate PPM and EOAi < 0.65 cm^2^ / m^2^ for severe PPM. It seems that in our study group who underwent isolated aortic valve surgery, the PPM incidence is similar to other clinics. It remains an usual phenomenon.

**Figure 3:**
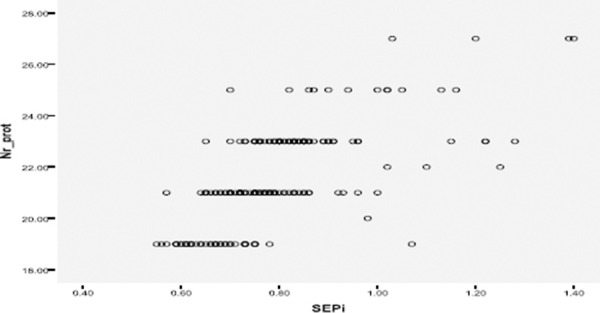
*Correlation between the number of prostheses and PPM. It is clear that the smaller the number, greater probability of occurrence PPM. Small prosthesis number is significant risk factor for the occurrence of PPM*.

The fact that this is common occurrence opened the debate about its impact on the results of aortic valve surgery. This debate is nowadays still open.

In our study, the majority of the indicators of early operative results such as mortality, low cardiac output, pulmonary complications, bleeding, atrial fibrillation and wound infection have higher incidence with the patient-prosthesis mismatch group compared with the group without this phenomenon. The overall mortality is 3.1% while in the group with severe PPM mortality was 5% in the group with moderate PPM 3.1% and for the group without PPM 2.4%.

Kaminishi et al, in a study, which involved 3609 patients with isolated aortic valve surgery, refer early mortality 2.2% for the group without PPM and 3.9% for the group with PPM. These are similar to our results. Despite mortality in the group with the PPM is greater, this difference is not significant. PPM importantly affects only the duration of stay in intensive care unit and respiratory assistance [8]. Jamienson et al in a series of 3343 patients concluded that early mortality independent risk factors are age, NYHAIII-IV, simultaneous coronary surgery, type of prosthesis, COPD, renal insufficiency and diabetes mellitus. PPM is not part of the risk factors for early mortality and even more the phenomenon does not affect long-term outcomes [9]. In addition, post-operative complications such as haemorrhage, stroke or complete atria-ventricular block, as well as in our study, are not affected by the presence of PPM [10]. There are also many smaller studies that support these results. In addition, PPM does not affect even the response of left ventricle muscle mass in the long-term follow-up [15]. Results of aortic valve surgery are satisfactory in the presence of 76% incidence of PPM [16].

At the same time, there are many studies, which refer PPM as an important risk factor in early results of aortic valve surgery [11, 13, 16-18]. Walther et al, in a study where were included 4131 patients, of which 1856 used mechanical prosthesis and 2275 biological prosthesis, analyzed the impact of PPM in early mortality of aortic valve surgery. Mortality for each group according to PPM degree was 5.2% in the group with severe PPM, 10.6% in the group with moderate PPM and 6.9% without PPM. Statistical analysis compared group with moderate PPM and without PPM and the difference was significant (p = 0.018). This group clearly concludes that PPM is an important risk factor in early mortality of aortic valve surgery based upon other factors such as emergency surgery, euro score greater than 10, age greater than 70 years old and simultaneous surgical procedures. This study recommended using information on specific prosthesis and specific data for each patient in order to avoid the PPM. In that context, various techniques can be used ranging from aortic valve annulus enlargement to surgery the aortic root [13].

Hernandez et al in a study very similar to this study, noticed that the mortality in the group with PPM was approximately 8.2%, while in the group without PPM, was 0.7%, with significant difference (p = 0.004), while for complications such as acute myocardial infarction, stroke, bleeding, post operative atrial fibrillation, pneumonia, the need for respiratory assistance, there is a tendency to have a higher incidence in the group with PPM and some of these differences are important and similar to this study. In conclusion, this group refers PPM as an important negative risk factor in early results in aortic valve surgery [16]. PPM is closely related with hospital mortality and cardiac events [11]. Supporting these findings is the group of Toronto who refer 8% mortality rate for patients with PPM and 5% in the group without MPP (p = 0.003). The last two groups referred to the moderate PPM and recommend the need to prevent the occurrence of PPM [17].

An interesting study that was published by Foster and colleagues, which refer that PPM increases operative mortality not significantly, but PPM impairs importantly muscle mass regress and in conditions when hypertrophy is an important risk factor for mortality, PPM may be a risk factor [14]. Patient-prosthesis mismatch should be analyzed together with patient characteristics. Urso and colleagues in a review associated with the effects of PPM concluded that severe PPM should be always avoided while moderate MPP can be tolerated except in cases of impaired cardiac function [19].

The controversy regarding the impact of early results of prosthesis- patient mismatch will probably continue. In this study, it seems that even PPM is a usual occurrence and does not influence negatively these results. Maybe in the future we will need to study this argument in special groups of patients such as in elderly patients or in patients with important left ventricle dysfunction. Furthermore, to determine the attitude towards PPM, it is very important to study this impact in long-term results of aortic valve surgery.

In conclusion, patient-prosthesis mismatch is a common occurrence in aortic valve surgery. This phenomenon does not affect the early results of aortic valve surgery.
